# Nutrient utilization, growth performance, and antioxidative status of Barki lambs fed diets supplemented with black (*Nigella sativa*) and rocket (*Eruca sativa*) seeds

**DOI:** 10.1007/s11250-024-04005-y

**Published:** 2024-05-10

**Authors:** Hassan Awny Fouad Rahmy, Reham Roshdi Ali El-Tanany, Wafaa Mostafa Ali Ghoneem

**Affiliations:** Animal Production Department, Faculty of Agriculture, Cairo University, Giza, 12613 Egypt

**Keywords:** *Nigella sativa*, *Eruca sativa*, Lambs, Growth performance, Antioxidant status

## Abstract

The current study aimed to determine the polyphenol compounds in *Nigella sativa* (NS) and *Eruca sativa* (ES) seeds, and evaluate the impact of their addition either as a sole additive or in combination on the growth performance, digestibility, some rumen and blood parameters and antioxidative status of Barki lambs. Forty-eight male lambs (27.18 ± 0.22 kg, 5–6 months), were divided into 4 balanced groups. The experimental diets were randomly distributed to the control group (CON); fed alfalfa hay plus concentrate feed mixture at a ratio of 30:70% without additives, while, NSD, ESD, and NESD groups: fed CON diet plus 2% NS, 2% ES or 1% NS + 1% ES, respectively as a ratio from total mixed ration (TMR). Results indicated that rutin and catechin were the most phenolic compounds observed either in NS or ES seeds. The NS and ES-supplemented groups recorded the highest (*P* < 0.05) values for dry matter digestibility, nutritive values, average daily gain, and the best feed conversion ratio. However, growth performance, nutritive value, and all nutrient digestibility except for dry matter were not significantly altered with the NESD group. Concentrations of ruminal NH3-N and TVFA were significantly (*P* < 0.05) reduced with the NESD group, with no significant differences in pH values among different groups. Values of blood parameters showed significant increases in WBCs, PCV, and T-AOC, and decreases in cholesterol, triglycerides, and MDA with the addition of NS and ES seeds or both. Therefore, the addition of NS and ES seeds is recommended to improve lambs’ health and antioxidant status.

## Introduction

Over many decades, several investigations have been done on the use of medicinal plants in ruminants’ nutrition. Black seed (*Nigella sativa*, NS); is one of these plant seeds that can be used effectively as a feed additive due to its high oil content, which contains diverse phytochemical compounds (Cherif et al. 2018b; Ahmed et al. 2021). Thymoquinone (TQ) is the major polyphenol in NS (Sahak et al., 2016), with many other compounds such as p-cymene, carvacrol, α-thujene, and β-pinene, which were noted to positively alter metabolism in the rumen and improve ruminant productivity (Odhaib et al. 2018b; Kabir et al. 2020). These active compounds were found to possess antioxidant, antimicrobial, anti-inflammatory, immunomodulator, and anticancer activity (Kooti et al., 2016; Ahmed et al., 2021). It was evident from a meta-analysis study conducted by Sadarman et al. (2021) that the supplementation of NS seeds in diets of small ruminants was effective in improving nutrient digestibility and growth performance of lambs. Furthermore, Obeidat (2020) showed improvements in the digestibility and growth performance of Awassi lambs when the diet was supplemented with NS meal. Moreover, NS supplementation either in the form of seeds or meals was observed to enhance animal immunity (Odhaib et al. 2018a; Talebi et al. 2021; Mohammed and Al-Suwaiegh 2023), and decrease levels of total lipids, LDL, cholesterol, and triglycerides in the blood (Tousson et al., 2011 and El-Hawy et al., 2018).

Rocket plant (*Eruca sativa*, ES) is one of the medicinal plants known under several names such as arugula or watercress. Several therapeutic uses have been confirmed for rocket leaves, oil, or seeds since ancient ages due to their diuretic effect, helping in digestion and bile secretion (Michael et al., 2011). Also, ES seeds were found to possess aphrodisiac, antifungal, and antibacterial properties (Bassyouni et al., 2022). A wide range of nutrients was observed in ES seeds such as vitamins A, E, C, proteins, glucosinolates (GLSs), flavonoids (mainly appin and luteolin), oils, and minerals such as Cu, Mg, Zn, Fe, and Mn (El-Nattat and EL-Kady, 2007; Abdul-Majeed and Taha, 2019). Gugliandolo et al. (2018) suggested that ES seeds can be used to prevent neurodegenerative diseases as a result of their flavonoids and GLS contents (Sarwar Alam et al., 2007; Bell et al., 2015 and Pagnotta et al., 2022), which exert anti-inflammatory and antioxidant activities (DinkovaKostova and Kostov, 2012; Orhan et al., 2015). Khaliq et al. (2021) mentioned that ES seeds contain napin protein, which can inhibit the growth of *Fusarium graminearum* and show antitumor activity. Meanwhile, the high concentration of erucic acid, an anti-nutritional factor, in ES seeds limits their uses as food or feed (Knutsen et al., 2016 and Kumar et al., 2023). However, the studies conducted on ruminants to evaluate the use of ES seeds in their diets were limited, Al-Fityin and ALSaig (2009) reported that the addition of 5% ES seeds as a percentage from concentrate feed mixture (CFM), improved the growth performance of Awassi lambs. Also, the inclusion of ES seeds in the diet of crossbred calves had no negative effect either on nutrient digestibility or growth rate (Das et al., 2003). Furthermore, dietary supplementation with ES seeds enhanced growth performance in rabbits (El-Nattat and EL-Kady, 2007), and reduced lipogenesis in broilers (Abou El-Maaty et al., 2021) and quail (Abdul-Majeed and Taha, 2019).

Therefore, the objectives of this study were to determine the polyphenols compounds in both NS and ES seeds, evaluate the impact of these seeds’ addition either alone or in combination on the growth performance, digestibility, some rumen and blood parameters and antioxidative status of Barki lambs.

## Materials and methods

The present study was carried out in February 2022 at the Agriculture Experimental and Research Station (Sheep and Goats Unit), Faculty of Agriculture, Cairo University. Chemical analyses were performed at the Animal Production Department Labs, Faculty of Agriculture, Cairo University, and Physiology Lab at Animal Production Research Institute, Egypt.

### Experimental design, animals, and rations

Forty-eight male Barki lambs with weights ranging from 22 to 30 kg (27.18 ± 0.22 kg), were divided into 4 balanced groups (12 animals each) according to the animal weight and age (5–6 months) for a 90-day growth trial. The growth trial was followed by 7 days for the digestion trial and collection of feces, rumen liquor, and blood samples. The experimental diets were randomly distributed to the experimental groups. Animals in the control group (CON) were fed alfalfa hay plus concentrate feed mixture (CFM) at a ratio of 30:70% without additives. While the other experimental animals were fed a control diet plus 2% Black seeds (NSD), 2% rocket seeds (ESD), or 1% Black seed + 1% rocket seeds (NESD) as a ratio from TMR.

The concentrate feed mixture consisted of 57% yellow corn, 10% soybean meal, 30% wheat bran, 1.6% limestone, 0.6% NaCl, 0.4% premix, 0.2% Na2Co3, and 0.2% anti-aflatoxin. Each 1 Kg of vitamins-minerals permix contains 8^6^ IU vit A, 4^5^ IU vit D_3_, 3^4^ mg vit E, carbonate calcium up to 1 Kg, and each 2 Kg of permix contains 5^4^ mg Zinc, 5^4^ mg Iron, 500 mg Iodine, 100 mg cobalt, 5^4^ mg manganese and 1^4^ mg copper. The chemical composition of seeds, CFM, alfalfa hay, and the experimental diets are illustrated in Table 1. All animal groups were fed rations at 4% of their live body weight to cover the total requirements as recommended by NRC (2007). Seeds of NS and ES were purchased from Harraz Company, Bab Elkhalq Square, Cairo, Egypt, and then mixed daily with CFM.

**Table 1:** Chemical composition of CFM, hay, NS and ES seeds, and the experimental diets (on a DM basis)

Item	Feedstuffs				Experimental diets*			
	CFM	Hay	NS	ES	CON	NSD	ESD	NESD
DM	94.24	90.76	94.72	95.45	93.20	93.23	93.24	93.23
**Chemical composition, % (DM basis)**								
OM	96.61	88.68	95.27	94.79	94.23	94.25	94.24	94.25
CP	13.12	16.80	22.15	24.42	14.22	14.38	14.42	14.40
CF	4.56	29.42	7.02	9.15	12.02	11.92	11.96	11.94
EE	1.99	4.25	28.76	25.81	2.67	3.18	3.12	3.15
NFE	76.94	38.21	37.34	35.41	65.32	64.77	64.74	64.76
Ash	3.39	11.32	4.73	5.21	5.77	5.75	5.76	5.75

CFM: Concentrate feed mixture. NS: black seeds. ES: rocket seeds. CON: control diet. NSD: control diet + 2% NS. ESD: control diet + 2% ES. NESD: control diet + 1% NS + 1% ES. *calculated. DM: dry matter. OM: organic matter. CP: crude protein. CF: crude fiber. EE: ether extract. NFE: nitrogen free extract

Animals were housed during the experimental period in individual pens. The experimental CFM and alfalfa hay (TMR) were offered for lambs 2 times per day at 7:00 a.m. and 4:00 p.m. The animals had free access to water and mineral blocks. Animal weights were recorded each 15 days and the feeding rates were adjusted. Feed intake was calculated daily for each animal as the difference between orts and the feed offered. Consequently, the values of feed conversion ratio (FCR) were estimated as feed intake (g)/gain (g).

### Blood sampling and analysis

On the last day of the growth trial, blood samples were collected before the morning feeding from all lambs via the jugular vein into 2 glass tubes containing EDTA anticoagulant (Ethylene Diamine Tetra Acetic acid) to perform analysis either on whole or plasma samples.

The first tube for each animal was directly transferred to the laboratory to initiate the hematological analysis. Red blood cells (RBCs), white blood cells (WBCs), packed cell volume (PCV), hemoglobin (Hb), neutrophil, lymphocytes, basophil, and eosinophil were estimated using a hematology analyzer (no: 51,619, Boule medical AB, Stockholm, Sweden). The other tube for each animal was centrifuged at 4000 rpm for 15 min. to separate blood plasma, then plasma was stored at -20 °C until further analysis.

The plasma concentrations of albumin, total protein, creatinine, liver function (aspartate aminotransferase (AST) and alanine aminotransferase (ALT)), cholesterol, triglycerides, and urea were done by the methods of Doumas et al. (1971), Tietz (2006), Reed (2013), Richmond (1973), Young (2000), Lespinas et al. (1989), in the same order. Globulin concentration was calculated as [total protein – albumin]. Plasma total antioxidant capacity (T-AOC) was analyzed according to Koracevic et al. (2001). Plasma malondialdehyde (MDA) was assayed as described by Satoh (1978).

### Digestion trial, feces, and feeds analysis

A digestion trial was conducted for 7 days (4 days for adaptation and 3 days for feces collection) on all lambs using the fecal bag technique (Velásquez et al., 2021; Ghoneem and El-Tanany, 2023). Bags were emptied daily before feeding (7:00 am and 4:00 pm). The daily collected feces were mixed for each bag, then sprayed with sulfuric acid (10%) and frozen. Representative samples of feces were pooled for each animal, and then samples were dried at 60° C/24 h and kept until chemical analysis after grinding. Nutrient digestibility was done using the method of acid-insoluble ash (AIA) as described by Lee and Hristov (2013) and calculated via the following equation:

1 $$\begin{array}{l}\text{D}\text{i}\text{g}\text{e}\text{s}\text{t}\text{i}\text{o}\text{n}\,\text{c}\text{o}\text{e}\text{f}\text{f}\text{i}\text{c}\text{i}\text{e}\text{n}\text{t}\\=100-\left[100\times \frac{\% idicator\,in\,feed}{\% indicator\,in\,feces}\times \frac{\% nutrient\,in\,feces}{\% nutrient\,in\,feed}\right]\end{array}$$

The chemical composition of feces and feed (CFM, hay, and seeds) samples were done according to AOAC (2016). The percentage of nitrogen-free extract (NFE) was calculated as [100- (CF% + CP% + EE% + ash%)], where CF, CP, and EE refer to crude fiber, crude protein, and ether extract, respectively.

### Rumen liquor sampling and analysis

Rumen liquor samples were collected, before the morning feeding, 3 and 6 h post-feeding, from all animals on the last day of the experiment using the stomach tube attached to an electric suction pump (Poolthajit et al., 2021). The first 50 ml of collected rumen liquor was excluded to avoid saliva contamination. After straining the liquor samples via 3 layers of cheesecloth, the pH values were directly determined using a digital pH meter (Hanna instruments Inc., Woonsocket, Rhode Island, USA, no: HI98103). Ammonia nitrogen (NH3-N) concentrations in strained samples were determined according to the method of Szumacher-Strabel et al. (2002). However, total volatile fatty acids (TVFA) in the acidified liquor samples with ortho-phosphoric acid were measured by the method of steam distillation as recommended by Wang et al. (2016).

### The quantitative analysis of phenolic compounds in seeds

Five grams of grinded samples either from NS or ES seeds were extracted by ethanol solution 50% (v/v) as described by Gueffai et al. (2022).

The phenolic compounds that existed in the experimental seeds (NS and ES) were identified using High-Performance Liquid Chromatography (Agilent 1260 infinity HPLC series, USA). The separation of phenolic compounds was carried out as described by Rashid et al. (2021). The wavelength of the detector was set at 280 nm. 20 µl of the seeds’ ethanolic solution was taken for injection. The column (Akinetex®, Phenomenx, USA), was operated at 30 °C.

### Statistical analysis

The current data (nutrient digestibility, rumen parameters, growth performance, and blood parameters) were analyzed by one-way ANOVA procedure using SAS (2009) via the following model: Yij = µ + Ti + eij.

Where: Yij = experimental observation, µ = general mean of treatments, Ti = effect of treatment, eij = experimental error. Duncan’s New Multiple Range Test was applied to compare the different treatment’s means.

## Results

### Phenolic compounds in *Nigella sativa* (NS) and *Eruca sativa* (ES) seeds

**Fig. 1:**
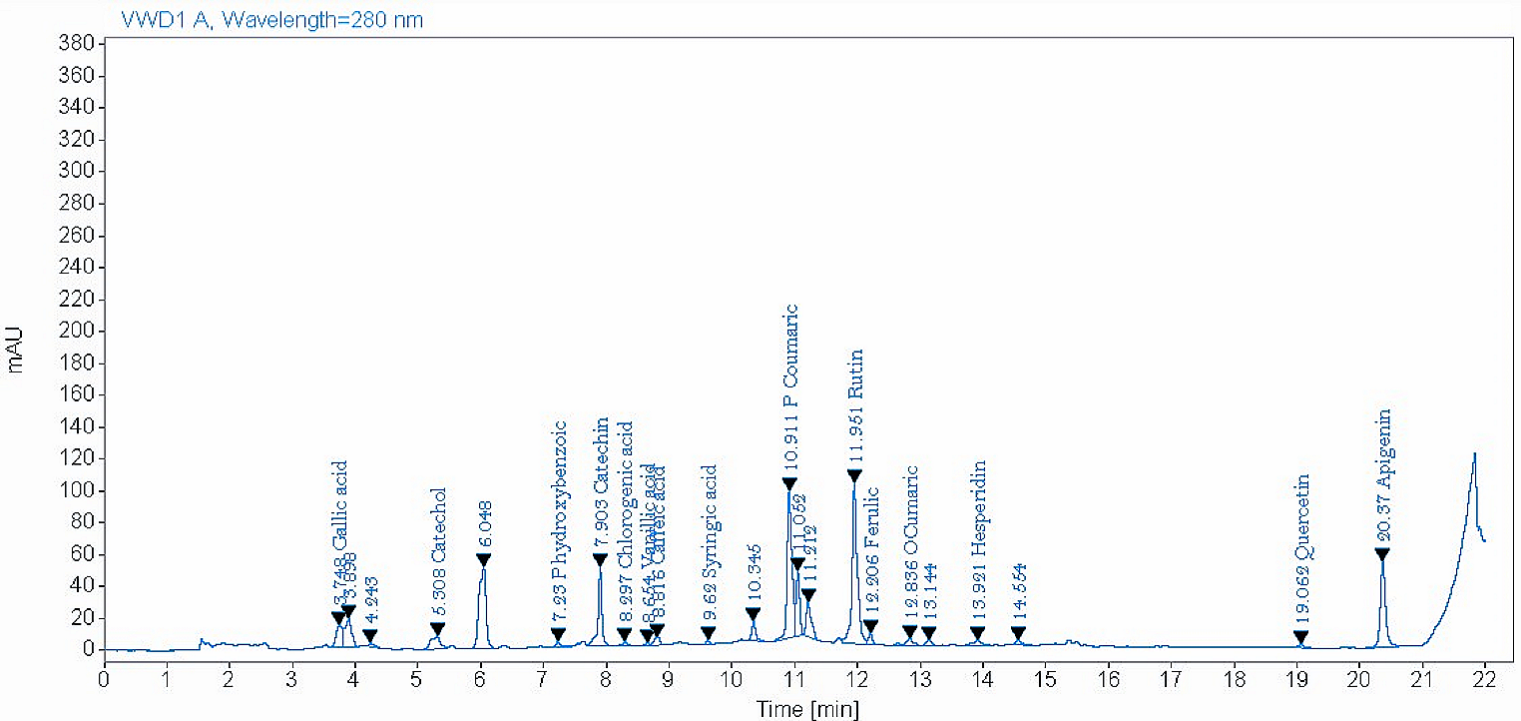
Phenolic compounds identified in rocket seeds (ES) by HLPC

**Fig. 2:**
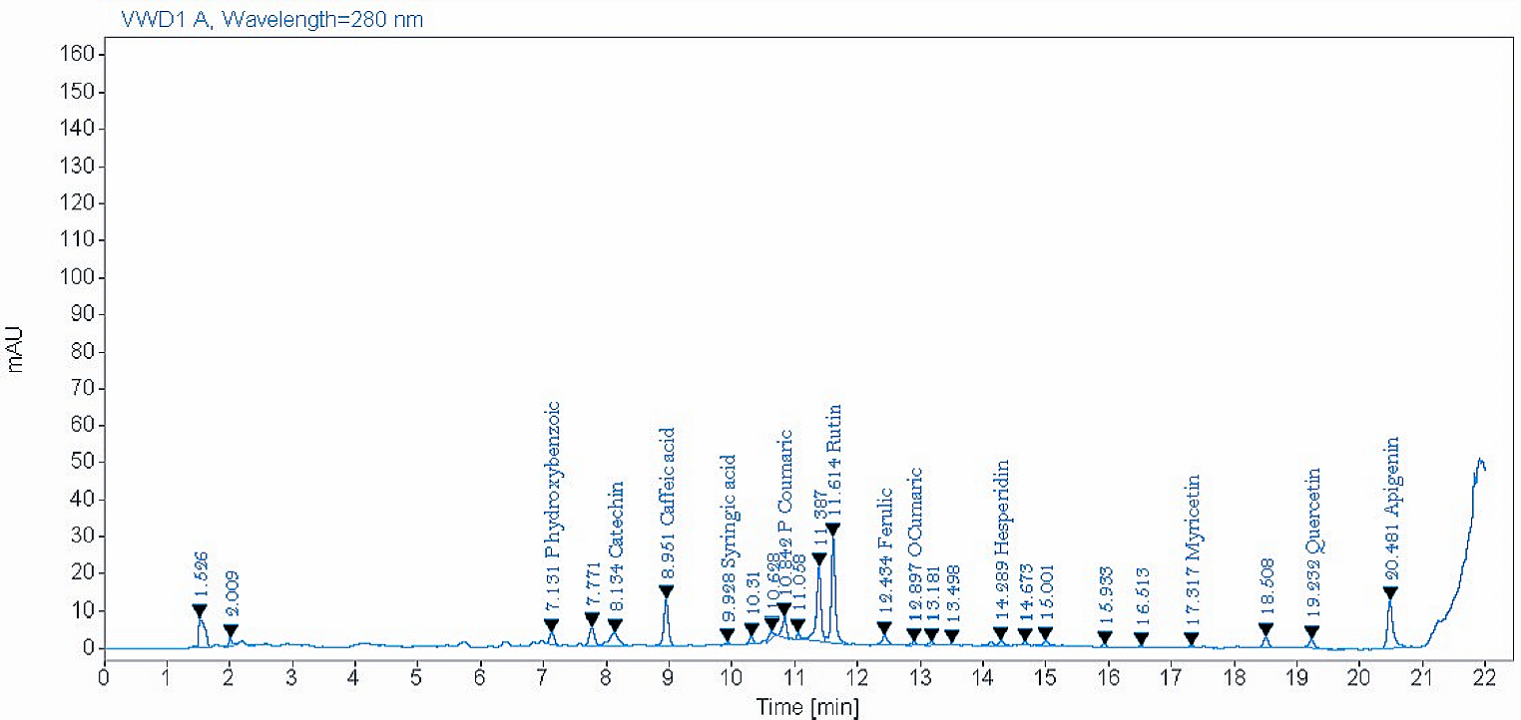
Phenolic compounds identified in black seeds (NS) by HLPC

The identified phenolic compounds by HLPC either in NS or ES seeds are presented in Table 2, Figs. 1 and 2. Data showed that 15 phenolic compounds were detected in ES extract compared to 12 compounds in NS extract out of 16 tested phenolic compounds. Catechin, rutin, p-coumaric acid, and catechol were the most phenolic compounds detected in ES. While, rutin, catechin, and quercetin were the most phenolic compounds observed in NS. Gallic acid, catechol, chlorogenic acid, and vanillic acid were found only in ES extract. However, myricetin was observed only in the NS extract. *Eruca sativa***(**ES) indicated high contents of the most detected phenolic compounds compared with NS.

**Table 2:** Phenolic compounds in NS and ES seeds

Phenolic compounds	ES seeds		NS seeds	
	RT (min)	Amount (mg/kg)	RT (min)	Amount (mg/kg)
Gallic acid	3.75	62.30	-	ND
Catechol	5.31	179.92	-	ND
P-Hydroxybenzoic	7.23	25.84	7.13	28.64
Catchin	7.90	674.12	8.13	117.93
Chlorogenic acid	8.30	33.23	-	ND
Vanillic acid	8.65	8.82	-	ND
Caffeic acid	8.82	25.09	8.95	47.71
Syringic acid	9.62	4.98	9.93	2.45
P-Coumaric	10.91	232.61	10.84	11.75
Rutin	11.95	535.55	11.61	121.96
Ferulic	12.21	73.12	12.43	43.41
O-Cumaric	12.84	14.95	12.90	2.00
Hesperidin	13.92	38.91	14.29	18.53
Myricetin	-	ND	17.32	1.90
Quercetin	19.06	90.37	19.23	111.90
Apigenin	20.37	30.02	20.48	7.76

ND: Not detected. ES: rocket seeds. NS: black seeds

### Nutrient digestibility and nutritive value of the experimental rations

Data associated with the effect of NS and ES seeds supplementation on nutrient digestibility and nutritive value are presented in Table 3. Positive significant (*P* < 0.05) effects of supplementation of each seed alone in lambs’ diets were observed on the digestibility of dry matter (DM). While it was significantly (*P* < 0.05) decreased with the combination of NS and ES seeds in the diet. On the other hand, the digestibility of organic matter (OM) and nitrogen-free extract (NFE) in NSD, ESD, or NESD diets were not significantly different compared with the control. Diet supplemented with black seeds alone (NSD) recorded the highest (*P* < 0.05) digestibility of crude protein (CP), with no significant difference among the diets containing rocket seeds (ESD and NESD) and control one. No significant difference among all experimental diets was observed in the digestibility of crude fiber (CF) and ether extract (EE). The highest nutritive value, as digestible crude protein (DCP) and total digestible nutrients (TDN), were recorded with NSD and ESD diets, with no significant difference between NESD and control diets.

**Table 3:** Effect of NS and ES seeds supplementation on nutrient digestibility and nutritive value of experimental diets

Item	Experimental groups				±SE	*P*-value
	CON	NSD	ESD	NESD		
**Apparent digestibility, %**						
DM	74.31 b	75.98 a	75.57 a	73.07 c	0.33	˂0.001
OM	76.68 ab	77.98 a	77.43 a	75.71 b	0.30	0.022
CP	74.95 bc	76.89 a	75.67 ab	73.93 c	0.34	0.004
CF	62.07	63.44	62.91	61.34	0.36	0.166
EE	80.40	82.43	81.24	82.16	0.46	0.417
NFE	79.24 ab	80.33 a	79.98 a	78.10 b	0.34	0.048
**Nutritive values, %**						
DCP	10.66 b	11.06 a	10.91 a	10.65 b	0.05	0.002
TDN	74.71 bc	76.55 a	75.92 ab	74.37 c	0.30	0.012

Significant differences (*P* < 0.05) exist among different letters in the same row

CON: control diet. NSD: control diet + 2% NS. ESD: control diet + 2% ES. NESD: control diet + 1% NS + 1% ES

DM: dry matter. OM: organic matter. CP: crude protein. CF: crude fiber. EE: ether extract. NFE: nitrogen free extract. DCP: digestible crude protein. TDN: total digestible nutrients

### Rumen liquor parameters

Data in Table 4 showed that neither seed supplementation nor sampling time had a significant effect on ruminal pH values. Similarly, the concentrations of both NH3-N and TVFA at zero sampling time were not significantly changed among different experimental groups. However, their values at 3–6 h post feeding were significantly (*P* < 0.05) reduced for the NESD group, with no significant difference among other groups and control.

**Table 4:** Effect of NS and ES seeds supplementation on some rumen liquor parameters of Barki lambs

Item	Sampling time, hrs.	Experimental groups				±SE	*P*-value
		CON	NSD	ESD	NESD		
pH	0	6.99	6.98	6.93	6.96	0.30	0.931
	3	5.48	5.38	5.45	5.42	0.31	0.755
	6	6.44	6.36	6.46	6.45	0.28	0.655
	Mean	6.30	6.24	6.28	6.28	0.22	0.831
NH_3_-N, mg/100 ml rumen liquor	0	5.73	5.89	5.68	5.71	0.15	0.971
	3	9.66 a	9.32 a	9.33 a	7.50 b	0.27	˂0.001
	6	7.15 a	7.03 a	7.06 a	5.67 b	0.20	0.001
	Mean	7.51 a	7.41 a	7.36 a	6.29 b	0.16	˂0.001
TVFA, mleq/100 ml rumen liquor	0	14.20	14.13	14.03	13.97	0.44	0.999
	3	18.50 a	18.87 a	18.10 a	15.80 b	0.38	˂0.001
	6	14.47 ab	14.97 a	14.10 b	12.30 c	0.32	˂0.001
	Mean	15.72 a	15.99 a	15.41 a	14.02 b	0.27	0.020

Significant differences (*P* < 0.05) exist among different letters in the same row

CON: control diet. NSD: control diet + 2% NS. ESD: control diet + 2% ES. NESD: control diet + 1% NS + 1% ES

### Growth performance

Data in Table 5 illustrated no significant differences among different experimental groups in initial, final body weight, and total body weight gain. However, lambs fed the NSD diet had the highest (*P* < 0.05) average daily weight gain (ADG), it was not significantly different from the ESD group compared with NESD or CON groups. In addition, the highest (*P* < 0.05) total dry matter intake (DMI) was observed with the NS-supplemented group (NSD), and the lowest value (*P* < 0.05) was recorded with the ESD group, with no significant difference between CON and NESD groups. Improvements (*P* < 0.05) in feed conversion ratio (FCR) values were detected with the addition of NS or ES seeds as a sole supplement compared with other groups.

**Table 5:** Effect of NS and ES seeds supplementation on growth performance of Barki lambs

Item	Experimental groups				±SE	*P*-value
	CON	NSD	ESD	NESD		
Initial body weight, kg	27.0	27.4	27.0	27.3	0.37	0.997
Final body weight, kg	42.3	45.1	43.7	43.1	1.01	0.196
Total weight gain, kg	15.3	17.7	16.7	15.8	0.74	0.703
ADG, g	170 b	197 a	186 ab	176 b	3.32	0.011
Total DMI, g	1355 b	1393 a	1325 c	1348 bc	6.87	˂0.001
FCR (DMI/ daily gain, g/g)	7.97 a	7.07 c	7.12 bc	7.66 ab	0.12	0.008

Significant differences (*P* < 0.05) exist among different letters in the same row

CON: control diet. NSD: control diet + 2% NS. ESD: control diet + 2% ES. NESD: control diet + 1% NS + 1% ES. ADG: average daily gain. DMI: dry matter intake. FCR: feed conversion ratio

### Blood biochemical, hematological parameters, and antioxidants status

Blood biochemical, hematological parameters, and antioxidant status of lambs in different experimental groups are presented in Table 6. The addition of NS or ES seeds either as a sole additive or in combination had no significant effect on concentrations of blood albumin, globulin, AST, ALT, urea, and creatinine. There was no significant effect of seed supplementation on blood total protein (TP) compared with CON, the highest TP value (7.11 g/dl) was recorded for the NSD group, and the lowest value (6.78 g/dl) was recorded for the NESD group. However, dietary supplementation with the current medicinal additives significantly (*P* < 0.05) reduced both levels of blood cholesterol and triglycerides. Regarding blood hematological indices, no significant differences were observed among different groups in red blood cells (RBCs), neutrophils, eosinophils, basophils, and hemoglobin (Hb). Diets containing NS seeds (NSD and NESD) significantly (*P* < 0.05) increased the count of white blood cells (WBCs), with no significant difference between the ESD and CON groups. Lambs-fed CON diet recorded the lowest values of packed cell volume (PCV), lymphocytes, and the highest monocytes compared with other groups. Data indicated that the addition of NS or ES seeds in lambs’ diet significantly (*P* < 0.05) increased total antioxidant capacity (T-AOC), and decreased (*P* < 0.05) malondialdehyde (MDA) concentrations compared with control.

**Table 6:** Effect of NS and ES seeds supplementation on Blood biochemical, hematological parameters, and antioxidants status of Barki lambs

Item	Experimental groups				±SE	*P*-value
	CON	NSD	ESD	NESD		
**Biochemical parameters**						
TP (g/dl)	6.91 ab	7.11 a	6.98 ab	6.78 b	0.45	0.038
Albumin (g/dl)	2.84	2.87	2.83	2.55	0.06	0.143
Globulin (g/dl)	4.07	4.24	4.15	4.23	0.30	0.190
AST (U/l)	71.92	69.55	69.07	70.15	2.15	0.978
ALT (U/l)	39.66	37.58	38.51	37.48	0.44	0.281
Cholesterol (mg/dl)	70.91 a	67.15 b	66.84 b	65.30 b	0.71	0.006
Triglycerides (mg/dl)	11.92 a	10.31 b	10.30 b	10.10 b	0.27	0.021
Urea (mg/dl)	22.08	21.74	22.23	22.54	0.75	0.990
Creatinine (mg/dl)	1.47	1.42	1.48	1.49	0.11	0.998
**Hematological indices**						
RBCs ×10^6^ cells/µl	9.54	10.36	10.33	10.22	0.15	0.174
Hb (g/dl)	9.45	10.11	10.19	9.99	0.14	0.276
PCV %	32.43 b	34.21 a	34.47 a	34.37 a	0.31	0.023
WBCs ×10^3^ cells/µl	9.71 b	10.58 a	10.32 ab	10.87 a	0.17	0.005
Neutrophil ×10^3^ cells/µl	3.39	3.65	3.68	3.72	0.11	0.785
Lymphocytes ×10^3^ cells/µl	5.42 b	6.16 ab	6.11 ab	6.36 a	0.15	0.012
Monocytes ×10^3^ cells/µl	0.67 a	0.56 ab	0.39 b	0.54 ab	0.04	0.040
Eosinophil ×10^3^ cells/µl	0.17	0.17	0.11	0.20	0.02	0.217
Basophil ×10^3^ cells/µl	0.06	0.04	0.03	0.05	0.01	0.458
**Antioxidants status**						
T-AOC (mmol/l)	0.81 b	0.98 a	0.95 a	0.96 a	0.02	0.040
MDA (nmol/ml)	3.32 a	2.48 b	2.66 b	2.52 b	0.12	0.008

Significant differences (*P* < 0.05) exist among different letters in the same row

CON: control diet. NSD: control diet + 2% NS. ESD: control diet + 2% ES. NESD: control diet + 1% NS + 1% ES

TP: total protein. AST: aspartate aminotransferase. ALT: alanine aminotransferase. RBCs: red blood cells. Hb: hemoglobin. PCV: packed cell volume. WBCs: white blood cells. T-AOC: total antioxidant capacity. MDA: malondialdehyde

## Discussions

Recent trends in animal research support using natural feed additives in animal diets due to their potential benefits either for animal performance or health. Black seeds (*Nigella sativa*, NS) and rocket seed (*Eruca sativa*, ES), are two examples of medicinal herbs that can be used in ruminant diets to promote animal performance and boost the animal antioxidant status and immunity (El-Gindy et al., 2020; Abdul Qaiyyum and Nergis, 2022).

### Phenolic compounds in *Nigella sativa* (NS) and *Eruca sativa* (ES) seeds

The current results indicated that only 12 and 15 phenolic compounds were identified in NS and ES, respectively by HLPC using 16 standard phenolic compounds. However, Toma et al. (2015) and Hannan et al. (2021) recorded 19 phenolic compounds in NS seeds by HLPC-UV-MS. In agreement with the current findings, Abdelkader et al. (2022) observed that rutin was the main phenolic compound in ES seeds that was identified by HLPC-UV. In contrast, Abd-Elsalam et al. (2021) indicated that long extraction of ES seeds (72 h) by ethanol increased the identified phenolic compounds by LC-ESI-MS to 39 compounds. These variations in phenolic compound content may be attributed to the maturity stage, growing region effect, processing methods, or isolation technique (Hannan et al., 2021). The most identified phenolic compounds in the present study either in NS and/or ES seeds were catechin, rutin, p-coumaric acid, catechol, and quercetin. In addition, important phenolic compounds such as gallic acid and chlorogenic acid were identified only in ES extract, whereas caffeic acid was identified in both NS and ES seeds. Several pharmacological properties were recorded for the flavonol rutin, such as antioxidant, anticarcinogenic, cardioprotective, and neuroprotective effects (Ganeshpurkar and Saluja, 2017). Also, Kheiry et al. (2019) recorded antioxidant, anti-UV damage, antiangiogenic, and antiplatelet activities for p-coumaric acid. Quercetin (flavonoids) was shown to protect cells against lung cancer, osteoporosis, and cardiovascular problems (David et al., 2016; Dabeek and Marra, 2019). Gallic acid and catechin (flavanols), and chlorogenic acid were found to have an anti-inflammatory, antioxidant, hepatoprotective effect and several pharmacological properties (Sidhu and Zafar, 2018; Bae et al., 2020; Yang et al., 2020). Similarly, Serra et al. (2021) recorded the antioxidant properties of caffeic acid.

### Nutrient digestibility and nutritive value of the experimental rations

The current data indicated the improvement in nutrient digestibility and nutritive value either significantly (*P* < 0.05) for DM and CP digestibility, DCP and TDN, or numerically (OM, CF, EE, and NFE digestibility) with the addition of black seeds alone (NSD). In agreement with that result, supplementation of NS seeds either in diets of Ossimi sheep (100 mg/h) or lactating buffaloes (12 g/h) increased the digestibility of DM, OM, CP, CF, and nutritive value as TDN and DCP (Zanouny et al., 2013 and Abo El-Nor et al., 2007, in the same order). Also, it was indicated that the inclusion of NS seeds in the diets of Barbarine lambs (12 g/d) significantly increased the digestibility of DM, OM, CP, and CF (Cherif et al. 2018a). Furthermore, El-Basiony et al. (2015) reported higher DM and EE digestibility for dairy goats received diets supplemented with 7.5 g NS seeds/h/d compared with control, while that supplementation had no significant effect on digestibility of OM, CP, CF, and NFE. In the same context, El-Naggar et al. (2018) noted increases in the digestibility of all nutrients and nutritive value (TDN and DCP) when NS oil was added at 0.1 or 0.2% of Ossimi lambs’ diet. Moreover, the meta-analysis conducted by Sadarman et al. (2021) revealed increases in the digestibility of DM, OM, EE, and CP with the addition of NS seeds in small ruminants’ diets. On the other hand, no effect on nutrient digestibility was detected when the diet of Dorper lambs was supplemented with 1% NS seeds (Odhaib et al. 2018a). Also, the addition of NS oil either in the diets of dairy buffaloes (Khattab et al., 2011) or Karadi lambs (Mustafa et al., 2022) did not affect the digestibility of DM, OM, CP, and CF.

The improvement in nutrient digestibility with the addition of NS seeds may be due to its stimulant effect on the digestive enzymes, activation of the microflora population, or slowing the rate of feed passage through the digestive tract (El-Saidy et al., 2004; Zanouny et al., 2013). Furthermore, Patra and Yu (2012), Griss et al. (2020), and Costa et al. (2021) confirmed the role of NS seeds in ruminants’ diet as modulators of rumen fermentation, which help in enhancing microbiota activity, enzyme secretion, digestibility, and absorption processes. In addition, Mohammed and Al-Suwaiegh (2023) showed significant increases in the total number of ruminal bacteria and protozoa when NS seeds were added at 10–20 g/kg of Ardi goats’ diet, which may explain the higher digestion coefficients. Also, it was indicated that the saponins content in NS seeds may result in more nutrient utilization due to their stimulant effect on organic matter fermentation (El-Basiony et al., 2015).

Regarding the effect of ES seed supplementation, the ESD diet recorded a significant (*P* < 0.05) increase in DM digestibility, with higher non-significant values for the other nutrients. However, Fayed and Azoz (2018) reported significant increases in the digestibility of CP, CF, EE, NFE, and nutritive value as TDN, with no significant effect on the digestibility of DM, OM, and DCP value when ES seeds were added at 1% of growing rabbits’ diet. Also, supplementation of rabbits’ diet with 3% ES seeds meal increased the digestibility of OM, CP, CF, EE, NFE, and values of TDN and DCP with no significant effect on DM digestibility (El-Nomeary et al., 2015). Furthermore, the replacement of 25 and 50% of mustard cake with ES oil cake in diets of crossbred calves had no significant effect on the digestibility of CP, CF, and EE. While, the digestibility of DM and OM were significantly increased only with 50% replacement (Das et al., 2003). Moreover, the digestibility of OM, CP, and NFE was not significantly influenced by replacing 50% of soybean meal with ES meal in rabbits’ diet (El-Tohamy and El-Kady, 2007). The relative improvement in nutrient digestibility and nutritive value with ES seed supplementation in the present study may be related to the flavonoids (such as carotenoids and isothiocyanate), vitamin C, and essential oil content in ES seeds which had a beneficial influence on digestive system activation (El-Tohamy and El-Kady, 2007).

The current findings demonstrated that nutrient digestibility and nutritive value were not significantly changed when diet supplemented with a mixture of NS and ES seeds (NESD), except for DM digestibility which was significantly (*P* < 0.05) decreased being 73.07% compared with control being 74.31%. NESD diet recorded the lowest values of nutrient digestibility and nutritive value compared with other diets (NSD and ESD). In matching with the current data, Odhaib et al. (2018a) found that supplementation of Dorper lambs’ diet with a combination of NS seeds and rosemary leaves decreased nutrient digestibility compared with control or diets supplemented with each of NS seeds or rosemary leaves alone. That result could be attributed to the higher content of total polyphenols in that diet, which may negatively affect fiber-degrading bacteria (*F. succinogenes* and *R. albus*) and total protozoa (Odhaib et al. 2018a). In addition, the reduction in fiber digestibility could be explained by the high polyphenols content especially tannin, which may bind the bacterial enzymes, prevent the microbial attachment to dietary fibers, or form complexes with cellulose, hemicellulose and pectin (Frutos et al., 2004). Furthermore, it was indicated that although high tannin content in feeds that bind with protein can increase the amount of rumen undegradable protein, it may also restrain the synthesis of microbial protein in rumen (Wang et al., 2022). Wanapat et al. (2008); Odhaib et al. 2018a); Kholif et al. (2021) suggested that the reducing effect of the medicinal plant’s mixture on digestibility compared with the sole supplement may be ascribed to a synergistic effect or a dose-dependent of these supplements.

### Rumen liquor parameters

In harmony with the present findings, non-significant effects on ruminal pH values were observed either at zero, 2, 6, or 12 h post-feeding when goats’ diet was supplemented with NS meal (Thayalini et al., 2018). In the same trend, the addition of 0.15 or 0.30% NS oil to the diets of Kardi lambs had no significant influence on pH values either before or 4 h after feeding (Mustafa et al., 2022). The same result was obtained by Abdullah and Farghaly (2019) when 33.3 or 66.7% of dietary cottonseed meal was replaced by NS meal in diets of growing lambs. In contrast, significant increases in ruminal pH were recorded with the supplementation of NS seeds either in the diets of Dorper lambs (Odhaib et al. 2018a) or Ardi goats (Mohammed and Al-Suwaiegh, 2023). The non-significant impact of NSD or ESD on both concentrations of ruminal NH3-N and TVFA, or the significant (*P* < 0.05) decrease in their values with the NESD group, may explain the current findings of ruminal pH. Moreover, the quantity of saliva is an important factor that affects the value of rumen pH (Thayalini et al., 2018), which plays a role in the rumen as a buffering capacity (Salem et al., 2013). An improvement in saliva production was reported as a result of high phenolic content in NS or ES seeds (Odhaib et al. 2018a).

Although rumen NH3-N concentrations at 0, 3, and 6 h post feeding or the mean values were not significantly different among NSD, ESD, and CON, there were slight non-significant decreases in their values with medicinal additives. In addition, the combination between NS and ES seeds (NESD) significantly (*P* < 0.05) decreased rumen NH3-N at 3, 6 h post feeding and the mean value compared with control. In matching with the previous results, Odhaib et al. (2018a) recorded significant decreases in rumen NH3-N either with 1% NS seeds alone or in combination with rosemary leaves. Also, rumen NH3-N concentration was decreased with NS oil supplementation (El-Naggar et al., 2018 and Mustafa et al., 2022). Contrarily, a significant increase in rumen NH3-N was reported with the addition of NS seeds in goats’ diets (Mohammed and Al-Suwaiegh, 2023). The reduction in rumen NH3-N due to the addition of NS, ES seeds, or their mixture in the present study, may be attributed to the high phenolic content in these medicinal additives (Odhaib et al. 2018a). Similarly, a relationship between phenolic compounds and the reduction in concentration of rumen NH3-N was indicated by Yang et al. (2007) and Wanapat et al. (2013), which consequently reduced protein degradation in the rumen. Frutos et al. (2004) explained the effect of phenolic compounds on rumen NH3-N by reducing the fractional rate of protein degradation. Mustafa et al. (2022) ascribed that reduction by the essential oils (EO) inhibitory effect on the deamination process, which could be a result of inhibiting rumen bacteria such as *Clostridium sticklansii* and *Prevotella anaerobius* (McIntosh et al., 2003). In the same line with the present findings, the addition of an essential oil mixture in sheep diets was found to decrease both forestomach digestion of protein and concentration of NH3-N in the rumen with an increase in CP flux to the duodenum, which could be explained by higher ruminal protein bypass (Lin et al., 2013).

The current observations stated that dietary supplementation either of NS or ES seeds alone had no significant effect on concentrations of rumen TVFA at all sampling times. However, the combination between NS and ES seeds significantly (*P* < 0.05) reduced TVFA’s concentrations. In the same line with the previous results, concentrations of rumen TVFA at different sampling times (0, 2, 6, or 12 h post feeding) were not significantly altered with the addition of 0.8, 1.6 or 2.4% NS meal in goats’ diet (Thayalini et al., 2018). Furthermore, the addition of NS powder had no significant effect on TVFA in the *in-vitro* study (Medjekal et al., 2017). Odhaib et al. (2018a) revealed a significant decrease in ruminal TVFA when the diet of lambs was supplemented with a mixture of NS seeds and rosemary leaves. The same authors suggested that a combination of more than one medicinal plant may increase the antimicrobial effect of these additives, which can inhibit rumen microbiota activity and decrease the fermentation process. In contrast, significant increases in ruminal TVFA were observed when NS meal replaced 33.3 or 66.7% cottonseed meal in the diets of growing lambs (Abdullah and Farghaly, 2019). Also, El-Naggar et al. (2018) recorded higher TVFA with the addition of 0.1 and 0.2% NS oil in the diets of Ossimi lambs.

### Growth performance

The improvement in values of ADG, total DMI, and FCR in the current study with dietary supplementation of NS seeds, are in accordance with findings obtained by Hammed and Amao (2022) and Mohammed and Al-Suwaiegh (2023) when NS seeds were added either to rabbits or Ardi goats’ diets, respectively. Similarly, ADG and FCR were enhanced with the addition either of NS seeds in rabbits or growing Zaraibi goats’ diets (Habeeb and El-Tarabany, 2012 and El-Gindy et al., 2020) or NS oil in diets of Ossimi lambs (El-Naggar et al., 2018) and buffalos’ calves (Khattab et al., 2011). Contrarily, no significant effect was reported on ADG and FCR (Odhaib et al. 2018a) or feed intake (Abo El-Nor et al., 2007) when NS seeds were added either to diets of Dorper lambs or lactating buffaloes, in the same order. Farhangi et al. (2016) explained the higher feed intake with NS seed supplementation by stimulating the secretion of thyroid hormones. Furthermore, the existence of phenolic compounds in herbal and medicinal plants (such as NS seeds), may stimulate the animal’s appetite (Odhaib et al. 2018a; Mohammed and Al-Suwaiegh 2023). The positive effect of NS seed supplementation on growth performance may be attributed to the high nutritious value of NS seeds (Sadarman et al., 2021). Several beneficial chemical constituents were reported in NS seeds such as alkaloids, flavonoids, essential oils, trace minerals, and amino acids (Ahmed et al., 2021), which are important for both the production and health of small ruminants (Lillehoj et al., 2018). Furthermore, the high content of polyunsaturated F.A in NS seeds such as linoleic, linolenic, and oleic acids, can be used by the animal as an energy source (Ramakrishna Rao et al., 2003). Moreover, stimulation of feed intake and nutrient digestibility with NS seeds addition as observed in the current study may confirm higher efficiency of nutrient utilization.

Regarding the effect of ES seeds addition in the present study, the improvement in ADG (numerically) and FCR (*P* < 0.05) compared with control agrees with results obtained when ES seeds were added to the diets of rabbits (Fayed and Azoz, 2018). Furthermore, the inclusion of ES meal significantly enhanced ADG and FCR either of crossbred calves (Das et al., 2003) or rabbits (El-Nattat and El-Kady, 2007; El-Nomeary et al., 2015). In addition, El-Badawy et al. (2018) reported that the addition of 1 or 2 mg ES oil/kg BW in diets of growing lambs improved ADG and FCR. The pervious observations may be ascribed to the ES seed’s content of aromatic compounds and essential oils. *Eruca sativa* (ES) seeds were found to be a rich source of vitamins (riboflavin, thiamine, niacin, pyridoxine, and folacin) and polyunsaturated fatty acids (Jaafar and Jaafar, 2019). Moreover, the role of ES seed oil in the alleviation of the toxic impact of aflatoxin B1 in the diets of rabbits (Hanafi et al., 2010) may be considered as an explanation. Also, improving the immunity of animals through increasing globulin, WBCs count and antioxidants in the current study (Table 6) can reflect on better growth performance. Abou El-Maaty et al. (2021) suggested that higher nutrient availability in diets containing ES seed meals may be the reason for growth performance enhancement. Whereas, Torres et al. (2020) concluded from a meta-analysis study that using EO at a level higher than 100 mg/kg of dietary DM reduced the ADG of sheep. In concordance with the present data, feed intake was significantly decreased when ES seeds were added to diets of New Zealand rabbits at 1% (Fayed and Azoz, 2018) or when ES cake was included in diets of crossbred calves (Das et al., 2003). In contrast, feed intake was significantly increased with ES supplementation (El-Nattat and El-Kady, 2007) or it was not significantly changed (El-Nomeary et al., 2015; El-Badawy et al., 2018 and Abou El-Maaty et al., 2021). Das et al. (2003) attributed the reduction in feed intake of crossbred calves to the lower palatability of ES cake, which may be due to the bitterness of erucic acid; the anti-nutritional compound in ES seeds (Knutsen et al., 2016). In addition, the pungency taste as a result of glucosinolates content in ES and their hydrolysis end products such as thiocyanate, isothiocyanate, and nitrile can be responsible for the reduction in feed palatability (Das et al., 2003). The effect of the combination of both NS and ES seeds on growth performance was similar to the control diet and matched with the findings of nutrient digestibility in the current study.

### Blood biochemical, hematological parameters, and antioxidants status

Blood biochemical parameters are considered indicators of the metabolism process and health status of the animal (Adeyemi et al., 2016). Also, these parameters can be used to evaluate the effect of medicinal herbs addition on animal physiology (Kim et al., 2013). The current data of blood TP were consistent with CP digestibility (Table 3), as the NSD group recorded the highest value and the NESD group recorded the lowest value. Mansour et al. (2013) indicated that higher blood TP with using NS meal in calves’ diet may be a reflection of enhancement in protein digestibility. Furthermore, it may be attributed to active compounds in NS such as thymoquinone, nigellone, and nigllen or essential amino acids content, which are necessary for protein synthesis (Amin and Hosseinzadeh, 2016).

However, no significant effect of medicinal seeds supplementation was detected on blood albumin and globulin, there was a non-significant improvement in globulin concentrations. These improvements in TP and globulin can serve as indicators of better immunity in the animal (Mansour et al., 2013). Gholamnezhad et al. (2015) reported immunomodulatory properties for NS seeds via increasing globulin and lymphocyte values (Ahmed et al., 2021). Moreover, higher blood immunoglobulin Ig (A, G or M) with NS seeds addition (El-Hawy et al. 2018; Odhaib et al. 2018a; El-Gindy et al. 2020), may reflect their immune-stimulatory properties, which can be ascribed to its anti-inflammatory, antibacterial and antioxidant effect (Ahmed et al., 2021). Also, NS seeds exhibited immunomodulatory effects due to the reduction in oxidative stress as a result of promoting T cells in the animal (Salem, 2005). Khattab et al. (2011) explained the enhancement in immunity with NS by their high content of Mg, Cu, Mn, Zn, Se, vitamins E, A, C, and folic acid. In parallel with the current observations, the addition of NS seeds in the diets of sheep and goats increased concentrations of blood TP and globulin either non-significantly (El-Hawy et al., 2018) or significantly (Habeeb and El-Tarabany, 2012; Mohammed and Al-Suwaiegh, 2023). In addition, Yavari et al. (2021) observed significant increases in blood TP, with no significant effect on albumin and globulin when lamb’s diet was supplemented with NS seeds. However, concentrations of TP, albumin, and globulin were not significantly altered by the addition of NS seeds in lambs’ and buffaloes’ diets (Odhaib et al. 2018a; Abo El-Nor et al. 2007, respectively). Also, blood TP and globulin were significantly unchanged with ES addition either in the form of seeds (Fayed and Azoz, 2018) or oil (Hanafi et al., 2010; Hafez et al., 2016). In contrast, Khalil et al. (2015) and El-Badawy et al. (2018) showed significant increases in TP and albumin with the addition of ES seeds or oil to fish or lambs’ diets, in the same order.

In coincidence with the present findings, no significant differences were observed in levels of blood ALT, AST, urea, and creatinine when NS seeds were added to diets of growing Zaraibi goats (Habeed and El-Tarabany, 2012), pregnant Barki ewes (El-Hawy et al., 2018) and Dorper lambs (Odhaib et al. 2018a). on the other hand, NS or ES seed supplementation significantly decreased values of ALT, AST (Yavari et al., 2021; Abou El-Maaty et al., 2021) and urea-N (Mohammed and Al-Suwaiegh, 2023). In the same trend, dietary supplementation with ES oil had no significant effect on blood ALT, AST, urea, and creatinine (Hanafi et al., 2010; Hafez et al., 2016 and El-Badawy et al., 2018).

In the present study, diets supplemented with NS, ES seeds, or both exhibited lower (*P* < 0.05) levels of blood cholesterol and triglycerides than control. These observations agree with results obtained by Zanouny et al. (2013), Fayed and Azoz (2018), and El-Gindy et al. (2020) when either NS or ES seeds were added to the diets of rabbits or lambs. Similarly, the addition of ES oil significantly reduced both levels of cholesterol and triglycerides (Hafez et al., 2016; El-Badawy et al., 2018 and El-Giziry et al., 2021). Contrarily, Mustafa et al. (2022) reported no significant effect on blood cholesterol and triglycerides with NS oil addition to the diets of Karadi lambs. Although cholesterol is important; because it is considered an essential component either in cell membrane structure or the synthesis of steroid hormones, there is a correlation between cardiovascular diseases and high cholesterol levels (Jeong et al., 2018). The hypolipidemic effect of NS may be due to the synergistic action of the thymoquinone content, its derivatives (thymol, thymohydroquinone, and dithymoquinone), soluble fibers (such as mucilage), flavonoids, sterols, high polyunsaturated fatty acids, and nigellamine (Ali and Blunden, 2003). Thymoquinone exhibited hypolipidemic action by decreasing cholesterol synthesis (Talati et al., 2009), reducing its absorption, increasing the production of bile acid, and increasing cholesterol excretion in feces (El-Gindy et al., 2020). El-Beshbishy et al. (2006) demonstrated that flavonoids can improve the efficiency of liver cells to reduce LDL cholesterol and increase its receptor number in the liver. In addition, the phenolic compounds in essential oils have the ability to reduce 3-hydroxy-3-methylglutaryl coenzyme A reductase, the key enzyme in cholesterol synthesis (Lee et al., 2004). In the same trend, ES seeds exhibited hypolipidemic action, which may be ascribed to the high content of unsaturated F.A (82% of oil) such as linoleic and linolenic (Singh et al., 2014), or as a result of glucosinolates that constrains lipid peroxidation (Alhila et al., 2022). Also, high vit. C content in ES may promote cholesterol conversion into bile acid, which results in reducing cholesterol levels in the blood (Hillstrom et al., 2003). Moreover, β-sitosterol in ES can reduce cholesterol absorption from the small intestine (El-Gengaihi et al., 2004). Furthermore, some active compounds in ES seeds showed hypolipidemic effects such as phenols, alkaloids, flavonoids, and turbines (Ahmed et al., 2016), and saponins (Zamani et al., 2007).

The current hematology parameters indicated significant (*P* < 0.05) higher values of WBCs, lymphocytes, and PCV, non-significant improvement in values of RBCs and Hb with diets supplemented with NS, ES, or both. Similarly, significant increases in RBCs, WBCs, Hb, PCV (El-Gindy et al., 2020), and lymphocytes (Mohammed and Al-Suwaiegh, 2023) were recorded when NS seeds were added to the diets of rabbits and goats, respectively. Also, the addition of ES seeds significantly increased RBCs, Hb, PCV (Shams Al-dain and Jarjeis, 2015), and WBCs (Khalil et al., 2015). Meanwhile, Odhaib et al. (2018a) noticed no significant effect of NS seed supplementation on the previous parameters. In addition, the replacement of 50% of soybean meal in the diets of New Zealand rabbits either with NS or ES meal did not significantly affect these hematological parameters (El-Nattat and El-Kady, 2007). These positive results in blood hematological indices may reflect the healthy status and high immune response of the animal (Khalil et al., 2015). Also, it was indicated that carotenoids in ES seeds may save phagocytic cells away from oxidative damage, increase B and T lymphocyte responses, and produce higher interleukins (Bendich, 1989). Furthermore, higher blood hematological parameters (Hb, RBCs, and PCV) may be a reflection of good iron percentage in ES seeds (Hassan et al., 2023). It was confirmed that thymoquinone content and the other active compounds in NS seeds may enhance the defense mechanism of the animal body against disease (Kanter et al., 2003). They also can improve the utilization of dietary nutrients, and increase the growth rate of the animals (Al-Beltawi and El-Ghousein, 2008), which is reflected in higher Hb, RBCs, and PCV (Shams Al-dain and Jarjeis, 2015). In addition, El-Hawy et al. (2018) suggested that the addition of NS seeds stimulates several hormonal factors that promote WBCs formation and increase their count.

The present data showed significant (*P* < 0.05) higher total antioxidant capacity (T-AOCA), and lower (*P* < 0.05) malondialdehyde (MDA) with NS and ES supplementation. These results are in accordance with those obtained by Hanafi et al. (2010), El-Badawy et al. (2018), El-Hawy et al. (2018), Fayed and Azoz (2018), Selim et al. (2019) and El-Gindy et al. (2020) when NS or ES either in form of seeds or oil were added to diets. Mohammed and Al-Suwaiegh (2016) demonstrated the positive effect of NS on ruminants’ production, health, and antioxidant status. Moreover, Habeeb and El-Tarabany (2012) and Abo-Zeina et al. (2013) showed that the high content of trace elements in NS seeds may improve the antioxidant status of the animals, and help them to resist the stress conditions. Mahmoud et al. (2021) confirmed the participation of NS seeds in cellular protection, as it is rich in antioxidant compounds, or through activation of catalase and T-AOC enzymes (Sultan et al., 2015). Furthermore, the active components in NS seeds such as catechin, chlorogenic acid, ferulic acid, hydroxybenzoic acid, kaempferol, sinapic acid and ρ-coumaric acid were found to exhibit antioxidant and ROS (reactive oxygen species) scavenging effects (Shin et al., 2013; Farhoosh et al., 2016; Zang et al., 2017; Sanjeev et al., 2019 and Parvizi et al., 2020). Also, thymoquinone, the major component in NS seeds, plays an important role as a scavenger of free radicals (Ardiana et al., 2020). In addition, NS seed supplementation reduced MDA level, the indicator of lipid peroxidation, as a result of decreasing hydrogen peroxide, superoxide, and hydroxyl radicals in the animal’s cells (Leong et al., 2013). It was indicated that thymoquinone has the ability to suppress non-enzymatic peroxidation of lipids in liposomes (El-Hawy et al., 2018). Fayed and Azoz (2018) reported a strong scavenger action of both ES leaves and seeds for free radicals, through increasing antioxidant molecules and enzymes which can reduce damage resulting from oxidation. The antioxidant effect of ES seeds may be due to erusin content, the main glucosinolate, which has the ability to protect cells from oxidation stress by preventing the accumulation of alkyl hydroperoxides and hydrogen peroxide in cells, or due to the increase in the antioxidant defense of cell via acting as sulforaphane precursor (Barillari et al., 2005).

## Conclusion

The present data demonstrated that nutrient digestibility and nutritive value were improved either with supplementation of NS or ES seeds alone, however NESD group was not significantly altered. NS-supplemented lambs recorded the highest ADG, total DMI, and the best FCR. Furthermore, the NESD group showed the lowest concentrations of ruminal NH3-N and TVFA, with no significant differences in pH values among all experimental groups. The addition of NS and ES seeds or both significantly (*P* < 0.05) increased values of blood WBCs, PCV, T-AOC, and decreased cholesterol, triglycerides, and MDA. Therefore, the addition of NS and ES seeds is recommended to improve lambs’ health and antioxidant status.

## Data Availability

The raw data for this research will be available from the corresponding author upon request.
